# Free cessation aids and enhanced support for smoking cessation in disadvantaged smokers: a qualitative study of patient and provider insights

**DOI:** 10.1017/S1463423626100942

**Published:** 2026-02-25

**Authors:** Paloma Vera, Maria Melchior, Djylal Badreddine, Marie-Noel Al Zayat, Gladys Ibanez, Melanie Böckmann, Fabienne El-Khoury

**Affiliations:** 1 Sorbonne Université, INSERM, Pierre Louis Institute of Epidemiology and Public Health, IPLESP, Social Epidemiology, Mental Health and Addictions, ESSMA, F75012, Paris, France; 2 University of Bremen: Universitat Bremen, Germany

**Keywords:** Nicotine replacement therapy, professional-patient relations, shared decision-making, smoking cessation, vulnerable populations

## Abstract

**Aim:**

To explore facilitators and barriers to smoking cessation among smokers experiencing socioeconomic disadvantage, from the perspectives of patients and healthcare providers (HP) participating in the STOP randomized controlled trial (STOP-RCT).

**Background:**

Smoking remains disproportionately prevalent among socioeconomically disadvantaged individuals, contributing to significant health disparities. The STOP-RCT evaluates a preference-based smoking cessation intervention offering free nicotine replacement therapy (NRT) and e-cigarettes to disadvantaged smokers.

**Methods:**

A qualitative study was conducted involving semi-structured interviews with 14 participants and 5 HP from the STOP-RCT. Data collection explored participants’ smoking cessation experiences, perceptions of the intervention, the quitting process, and the factors that influence cessation. Thematic analysis was used to analyse the transcribed data. Themes were categorized into structural and individual factors, refined iteratively, and supported by illustrative quotes.

**Findings:**

Four key facilitators were identified: (1) longer consultations enabling tailored support; (2) regular follow-up promoting patient engagement; (3) immediate and free access to NRT and carbon monoxide (CO) monitoring, reducing financial and practical barriers while providing feedback; and (4) shared decision-making, strengthening trust and improving the fit of support. These findings highlight the importance of addressing both treatment approach (contextual) and interpersonal factors for this population. Considering these elements may help adapt cessation programmes to the specific difficulties and needs of patients with low socioeconomic position, thereby reinforcing treatment adherence and improving effectiveness.

## Introduction

Smoking is a leading cause of preventable death worldwide (Reitsma *et al.*, 2021) and disproportionately affects individuals from lower socioeconomic positions (SEP) (Casetta *et al.,* 2017). Although smokers across all social groups intend to quit at similar rates, those with lower SEP face greater barriers to cessation (Kotz and West, 2009; Bauld *et al.*, 2010; Brown, Platt and Amos, 2014; Hill *et al.*, 2014). In France, for instance, lower educational attainment and unemployment are strongly associated with a higher prevalence of daily smoking. (Pasquereau *et al.*, 2024). Evidence from multiple international studies (Hiscock *et al.*, 2012) shows that disadvantaged smokers encounter several significant barriers to cessation. They inhale more intensely, which results in a greater extraction of nicotine and tar from each cigarette. A lack of social support further complicates this process, as their networks frequently include a high proportion of smokers, reinforcing the behaviour and limiting encouragement to stop. Individuals may also face diminished motivation to quit, often due to prioritizing immediate concerns over long-term health risks. These challenges are exacerbated by psychological factors, including lower self-efficacy and elevated life stress, which collectively hinder sustained cessation efforts.

Healthcare providers (HP) play a crucial role in supporting smoking cessation efforts, especially among disadvantaged populations. In France, smokers can consult various, including general practitioners (GP), addiction specialists, and tobaccologists (specialists trained in tobacco dependence). Eligible professionals for a specialization in ‘tobaccology’ include doctors, psychologists, pharmacists, dentists, nurses, midwives, and physiotherapists (Haute Autorité de Santé, 2023). As primary care physicians, GP are well-positioned to address smoking cessation within routine care. They can provide motivational support, cessation advice, and prescribe NRT or medication (Raw, McNEILL and West, 1998; Smith, Hill and Amos, 2020). By contrast, addictologists and tobaccologists generally provide more specialized and extended consultations.

However, smokers with lower socioeconomic status often face barriers to accessing these services, leading to underutilization and poorer outcomes (European Commission *et al.*, 2018). Personalized support that reflects patient preferences offers a promising approach to mitigating these disparities, as it has been shown to be more effective than standardized methods due to the strong influence of beliefs and expectations on treatment outcomes (Bowling & Ebrahim, 2001).

In line with this approach, our study examines, our study focuses on the STOP (Sevrage Tabagique à l’aide d’Outils dédiés selon la Préférence: Smoking cessation using preference-based tools) randomized controlled trial (RCT). STOP is a preference-based smoking cessation programme designed to enhance smoking cessation success rates among smokers with low SEP. It offers tailored cessation aids, combined with professional guidance and comprehensive support (El-Khoury *et al.*, 2021).

Although social support has been consistently linked to successful smoking cessation (Yang *et al.*, 2013; Soulakova *et al.*, 2018), most research emphasizes the importance of family and community networks (DiMatteo, 2004). The patient–HP relationship has received less attention, despite evidence that medical support strongly shapes engagement and treatment attitudes (Matusitz and Spear, 2014; Jaworski, 2015). Effective communication and regular follow-up build trust and ensure that care meets individual needs (Kaba and Sooriakumaran, 2007), especially in addiction treatment (Miller and Moyers, 2015). In this context, shared decision-making (SDM) can empower patients, especially those often excluded from medical decisions, by tailoring care to their needs (Grabinski *et al.*, 2018). Through clear language, accessible formats, and inclusive practices, SDM can help reduce health inequalities and improve cessation outcomes (Durand *et al.*, 2014).

Despite the acknowledged importance of the HP-patient relationship in healthcare, its specific role in smoking cessation remains understudied, particularly from the perspectives of both HP and patients. This gap in the literature limits our understanding of the interpersonal and contextual factors that contribute to successful cessation. To address this gap, this qualitative study explores HP and patients’ perspectives on how STOP’s components and interactive approach shape the cessation process.

## Methods

### Study design: the STOP randomized controlled trial

A sample of patients and HP participating in the STOP trial, a pragmatic multicentre RCT, was recruited for this qualitative study. The trial was conducted in 17 centres across five French regions: Île-de-France (Paris region), Provence-Alpes-Côte d’Azur, Pays de la Loire, Hauts-de-France, and Auvergne-Rhône-Alpes. In the RCT, smokers with low SEP, recruited by GP or tobacco-cessation specialists in medical settings, were randomly assigned to either the intervention or control group (El-Khoury *et al.*, 2021). The control group receives standard care, which reflects each HP usual practice. This generally includes motivational interviewing and brief advice to quit smoking, as well as the prescription of cessation treatments such as NRT, for which costs can be reimbursed. The intervention group receives a choice of various NRT (patches, gums, inhalers, sprays, tablets, etc.) and/or electronic cigarettes with ‘e-liquids’ all provided immediately and free of charge, to support quit attempts, according to their preference. Furthermore, the STOP RCT questionnaire incorporated motivational interviewing techniques typically employed by tobaccologist in smoking cessation interventions. As a result, GP participating in the STOP RCT were able to spend more time per consultation, allowing them to focus solely on smoking cessation. Follow-up assessments were conducted over a 6-month period, specifically at 10 days, and in the first, third and sixth months.

### Data collection

Data collection was conducted between March and July 2024. A qualitative descriptive design involving semi-structured interviews was employed. Interviews were conducted using a guide in French (Barriball and While, 1994). Data were collected via telephone (*n* = 9), video communication (*n* = 4), or in person interviews (*n* = 6) at the participating study centres (hospital outpatient clinics and community health centres), based on participant preference. All sessions were audio-recorded, transcribed, and anonymized and pseudonyms were used in the results section to present quotes and ensure confidentiality. Patients were encouraged to express themselves freely, with further probing as necessary. Interviews lasted between 35 and 110 minutes. Field notes were recorded both during and immediately following each interview to capture contextual details, non-verbal cues, and emergent themes.

Interview guides included thematic questions tailored to participants (smokers or HP) and their specific characteristics. Smokers were asked about smoking history, quit attempts, perceptions of cessation treatments, and experiences with medical support. HP were queried about developing smoking cessation consultations, the HP-patient relationship, and perspectives on the smoking cessation process. This guide was iteratively refined throughout the data collection process, based on insights gained from initial interviews, to ensure relevance and depth of inquiry.

Additionally, an exploratory observation was conducted during support group sessions with smokers and former smokers. This involved open memo writing guided by a thematic grid, capturing the context, vocabulary, and needs of individuals trying to quit.

The interviews were conducted by two master’s-level researchers (PV, female, and DB, male), both holding Master’s degrees in Sociology and possessing prior experience in qualitative fieldwork. They were part of the research qualitative team of STOP ECR, and their interests aligned with the broader objectives of the study, namely to explore the experiences of low SEP smokers participating in a cessation programme. At the outset, several thematic domains were considered potentially relevant, including the role of social relationships and environment, the perceived impact of the programme characteristics, and the patient-HP relationship.

One initial assumption was that the interaction between patients and HP might play a central role in shaping participants’ experiences. However, the interviews were conducted using a flexible framework, with no single aspect prioritized a priori. To deepen our understanding, we subsequently decided to conduct interviews with HP as well, despite this not having been initially planned.

The researchers had no prior relationship with any of the participants. All participants provided informed consent. At the beginning of the interview, the participants were informed that the study aimed to explore their experiences of smoking cessation support. This included how they managed the quitting process, the social aspects of this experience, their perceptions of the support they received and their experience of the HP-patient relationship. No non-participants were present during the interviews. The study was reported in accordance with the Consolidated Criteria for Reporting Qualitative Research (COREQ) checklist (Tong *et al.*, 2007), as recommended by the EQUATOR Network.

### Participants

For this qualitative analysis, we interviewed both smokers (participants) and HP involved in the STOP trial. To explore the role of medical support in smoking cessation and the impact of specific STOP programme features, we employed purposive sampling. This strategy aimed to recruit participants and HP likely to provide rich insights into the study’s focus. We sought a diverse sample considering sociodemographic characteristics, smoking habits, and HP roles to comprehensively understand the research questions (Table 1).

**Table 1.** Characteristics of participants in our study (March–July 2024, *n* = 19)

	Number (%)
**Sex**	
Female	14 (74%)
Male	5 (26%)
**PATIENTS**	**14 (74%)**
**Age**	
20–29 years	1 (7%)
30–39 years	2 (14%)
40–49 years	2 (14%)
50–60 years	3 (21%)
+ 60 years	6 (43%)
Range 21–69 years; Median 53 years	
**Arm**	
Standard care	7 (50%)
Intervention	4 (29%)
Not concerned	3 (21%)
**Follow-up**	
Completed	6 (43%)
In progress	3 (21%)
Not started	3 (21%)
Dropped out	2 (14%)
**HEALTHCARE PROFESSIONALS**	**5 (26%)**
**Profession**	
General practitioner	2 (40%)
Nurse	2 (40%)
Tobaccologist	1 (20%)
**Specialist in tobacco cessation**	
Yes	3 (60%)
No	2 (40%)

We included individuals who completed the 6-month follow-up (*n* = 6), were still being followed-up (*n* = 3), did not start (*n* = 3), or dropped out (*n* = 2). Both participants randomized to the standard care (*n* = 7) and intervention group (*n* = 4) were represented. The sample encompassed participants who achieved their goals (reduction or cessation) and those who did not. Participant ages ranged from 21 to 69 years, with recruitment across Île-de-France, Nouvelle-Aquitaine, Rhône-Alpes, and Nord-Pas-de-Calais regions.

Five interviews were conducted with HP, including specialist smoking cessation nurses (*n* = 2), a tobaccologist (*n* = 1), and GP (*n* = 2) Three of the professionals (60%) had completed a formal specialization in tobacco cessation (Table 1). These professional categories were chosen because, in France, they are authorized to prescribe NRT and their direct involvement in the STOP trial. The total sample size (*n* = 19) was considered adequate to ensure participant diversity, and allow for an in-depth analysis.

### Data analysis

The recorded interviews were transcribed and analysed using a hybrid approach combining inductive and deductive thematic (Patton, 1999) methods inspired by Braun and Clarke’s framework and elements of content analysis (Braun and Clarke, 2006). This process began with an iterative process of reading and re-reading transcripts to familiarize the researchers with the data (Thomas, 2006).

To guide the analysis, an initial coding framework was developed, with a priori codes informed by the hypothesis that both structural (treatment approach) and provider-patient relation (interpersonal) factors play significant roles (Kirk *et al.*, 2015). The process followed a vertical approach, where observations and interpretive notes were documented in the margins during a systematic reading (Crabtree and Miller, 1991). Major themes within each interview were identified through *open coding*, guided by principles from Grounded Theory (Corbin and Strauss, 1990), involving the systematic identification, analysis, and summarization of recurring themes. Emergent codes were generated inductively to capture unanticipated patterns and insights, while individual profiles were created to synthesize essential ideas from each interview. Subsequently, axial coding (Corbin and Strauss, 1990) was employed to reorganize and connect initial codes into categories and subcategories, allowing for an in-depth exploration of interpersonal factors. Finally, a thematic analysis was conducted to identify overarching themes across all interviews. This iterative process enabled the identification of key concepts through a systematic review of initial codes and themes, exploration of their relationships, and refinement of overarching themes through further analysis (Nowell *et al.*, 2017). The resulting themes were organized into two primary categories: treatment approach factors and interpersonal factors. Concepts and categories were further developed into sub-themes and key themes, involved constructing a thematic and theoretical framework, supported by transcripts illustrating each theme. Finally, categories and sub-themes were refined and validated in the field to accurately and comprehensively capture participants’ experiences and perspectives (Table 2). To protect participant anonymity, all names used in this manuscript are pseudonyms.

**Table 2.** Description of themes and sub-themes with quotes

Themes	Sub-themes	Definition	Quotes	Frequency of occurrence n (%)
**Treatment Approach**	Extended consultation time	consultations dedicate exclusively for tobacco cessation and more extended	‘It was a long time ago… It’s very different in a way… He doesn’t spend as much time and that’s for sure and not all doctors take the time, I think, to talk to their patients, um! It could… The GP could do it, but do they have the time? And would they take the time? I don’t know. […]’(Patient 5, intervention group, achieved complete cessation, follow-up completed)	8 (42%)
			‘Exactly, it improved the relationship very significantly and in that the study was very enjoyable. And then there was the fact that the funding allowed enough time to be dedicated to prevention. And it was also very nice to have dedicated time for a single purpose. In general practice, we often have two, three, four, five or more [purposes]. And here, having enough time, dedicated to one reason, was also very pleasant’. (Doctor 2, GP)	
	Ongoing follow-up and maintenance of treatment regularity	Regular follow-up sessions and continuous support to monitor progress, manage side effects, and sustain treatment adherence	[Did the follow-up help?] ‘Yes. It was especially the part where she asked me questions […]. They always have time to answer my questions, to reassure me, to encourage me: ‘Ah, you see, it’s not bad. We can carry on like this, we’ll manage to get this. In time, you‘ll see’. It’s a bit like support, maybe emotional support. I think it’s important to give up moral support, because that’s when it’s easiest to start again, I think’ (Patient 11, standard care group, reduced consumption, follow-up completed).	16 (84%)
			‘There was a moment when I wanted to stop everything, but I knew I’d made an appointment and I said to myself: ‘no, you’re not going to cancel, it’s not right at all, go on, you’re going’. And then it helped, it was good to meet the doctor. No, no, I think follow-up is really important […]’(Patient 1, standard care group, reduced consumption, follow-up ongoing)	
	Direct access to treatment	On-site availability of smoking cessation options facilitating timely initiation and patient choice	‘Oh well, completely, it would have been much easier. I would have had them. I would have had them at home and I certainly would have tried […], and I think I would have been less scared. It would have engaged me straight away’ (Patient 6, standard care group, treatment discontinued, no reduction or cessation).	9 (47%)
			‘No, I was pleasantly surprised because I was really given all the means that existed to help me. Afterwards, it was up to me to choose, so yes, when you don’t necessarily have the resources, well… I mean, if I’d wanted to, I’d have bought one, but having it on hand, at least you don’t have that barrier, you’ve got it on hand and that’s it. It’s like an extra support, in fact’. (Patient 5, intervention group, achieved complete cessation, follow-up completed)	
	CO Breath Test Monitors	Use of CO monitors to provide objective feedback on smoking behaviour, raise awareness of risks, and motivate cessation through visible progress tracking	‘Yes. In fact, I didn’t use it before the STOP program, and I was wrong, because in fact, I realized by using it, it had a really important impact to realize visually the intoxication we had, if I put myself in the patients’ shoes […]’(Doctor 2, GP)	7 (37%)
			‘The doctor makes me blow into something to see where I’m at. Last time it was 15, while the one before that was 16. It’s still red, you see. But next time I’ll surprise her and it’ll be yellow. And until it turns green’. (Patient 8, intervention group, reduced consumption follow-up ongoing)	
**Interpersonal Factors**	Shared Decision-Making	Collaborative process where patients and providers jointly make treatment decisions, integrating medical expertise with patients’ preferences and experiences.	‘Then, what can promote the doctor-patient relationship? In my opinion, it’s probably a model that’s not too paternalistic, a model where decisions are shared, where the issues are explained and shared, and so it’s the patient who takes ownership of the problem’ (Doctor 2, GP).	13 (68%)
			‘I was thinking about it, the commitment, what suited me, the choice she proposed, and then I got all the positive and negative information. And I felt more like I’d learned… When I made my choice to vape, I had the confidence, all the medical information she gave me, the trust, our choices, the extension of my choice… And that’s why it really took off for me, the trigger to stop.’ (Patient 4, intervention group, achieved complete cessation, follow-up completed)	
	Recognizing of tobacco use as a disease	Framing smoking as a medical condition of addiction rather than a matter of willpower, enabling patients to accept treatment and anticipate challenges	‘And then, his [the HP] explanation of addiction, of the phenomenon of addictology, to understand at some point that it’s not just weakness, but that it can also be a disease to find yourself addicted to something […]’(Patient 3, standard care group, achieved complete cessation, follow-up completed)	14 (74%)
			[…] ‘I present it as a chronic illness, and it’s precisely the illness of loss of control, what they call lack of willpower. I call it loss of control. So I’m also changing the way they talk. […] They need to feel less guilty, and that’s important’. (Doctor 1, GP).	
	Awareness of the need for a specific type of treatment	Understanding the necessity of personalized smoking cessation interventions, including proper dosing, treatment type, and adherence for effective outcomes’.	‘And then, we take the whole history of what they’ve done, stopped, not stopped, what may have worked, what may not have worked. After that, we’re careful, because patients often tell us: ‘I’ve already tried to quit with my GP (or with anyone else) and patches don‘t work’. So why not? Because when you teach yourself, they only use one patch, and as I see it, you need several. So we’ll explain, we’ll actually say: “I told you to under-dose, that’s why it didn’t work”. We can try again, at the right dose, and so on. […]’(Doctor 3, tobaccologist)	14 (74%)
			‘Ah yes, reassure them, show them that it’s not…. it’s not drugs, it’s just a treatment to help them at some point to quit smoking, and that they’ll be able to cut down and stop […]’(Nurse 1)	
			‘I thought it was like smoking, actually [the electronic cigarette]. So I thought, well, cigarette or vaporizer, it’s the same thing, so I might as well keep cigarettes. But not at all […] But I thought the electronic cigarette was the same thing as cigarettes, but it’s not.’ (Patient 7, intervention group, reduced consumption; follow-up ongoing)	

### Ethical considerations

The study was approved by the ‘Île-de-France II’ Institutional Review Board on 8 September 2020 (CPP Île-de-France II; Ref No: 20.01.31.65528 RIPH2 HPS).

## Results

Analysis of collected narratives revealed key factors influencing smoking cessation, which clustered into two overarching themes: the treatment approach (encompassing elements such as the type and intensity of counselling, and the use of aids like NRT and e-cigarettes) and the interpersonal factors, referring to the interaction between HP and patient. Participants were recruited until no new relevant insights appeared, which occurred after 19 interviews, indicating that thematic saturation had been achieved. The diversity within the sample, considering age, sex, and experiences with smoking cessation interventions, ensured that multiple perspectives were captured.

### The treatment approach

#### Extended consultation time

The STOP study has facilitated the implementation of medical appointments dedicated exclusively to helping patients quit smoking, particularly among GP, thereby increasing the time allocated for treatment discussions:

Exactly, it improved the relationship very significantly and in that the study was very enjoyable. And then there was the fact that the funding allowed enough time to be dedicated to prevention. And it was also very nice to have dedicated time for a single purpose. In general practice, we often have two, three, four, five or more [purposes]. And here, having enough time, dedicated to one reason, was also very pleasant. (Doctor 2, GP)

The impact of the timing of appointments was also mentioned by some patients. Amelia, who had successfully quit smoking with the help of her tobaccologist, wanted to understand why her previous attempt had failed despite the support of her GP:

It was a long time ago… It’s very different in a way… He doesn’t spend as much time and that’s for sure and not all doctors take the time, I think, to talk to their patients, um! It could… The GP could do it, but do they have the time? And would they take the time? I don’t know […] (Patient 5, intervention group, achieved complete cessation, follow-up completed)

### Ongoing follow-up and maintenance of treatment regularity

These appointments were accompanied by regular follow-up and ongoing communication between visits to address emerging challenges. These in-between sessions made it possible to monitor treatment progress and manage adverse effects of the smoking cessation process through counselling and treatment adjustments. Moreover, they provided the necessary moral and psychological support needed to address the difficulties associated with the social and psychobehavioural dependencies related to cigarette consumption.

[Did the follow-up help?] ‘Yes. It was especially the part where she asked me questions […]. They always have time to answer my questions, to reassure me, to encourage me: “Ah, you see, it’s not bad. We can carry on like this, we’ll manage to get this. In time, you’ll see.” It’s a bit like support, maybe emotional support. I think it’s important to give up moral support, because that’s when it’s easiest to start again, I think’ (Patient 11, standard care group, reduced consumption, follow-up completed).

The follow-up also helped patients maintain regularity, which motivated them to adhere to the treatment plan and avoid premature discontinuation:

There was a moment when I wanted to stop everything, but I knew I’d made an appointment and I said to myself: ‘no, you’re not going to cancel, it’s not right at all, go on, you’re going’. And then it helped, it was good to meet the doctor. No, no, I think follow-up is really important […] (Patient 1, standard care group, reduced consumption, follow-up ongoing)

### Direct access to treatment

In addition, participants in the intervention group received smoking cessation treatment on-site during STOP appointments. This direct access made it easier for patients to choose treatment according to their preferences. Some patients reported that they appreciated being able to try different options and decide without being limited by cost. They also noted that this approach shortened the time between the decision to quit smoking and the initiation of the cessation process:

No, I was pleasantly surprised because I was really given all the means that existed to help me. Afterwards, it was up to me to choose, so yes, when you don’t necessarily have the resources, well… I mean, if I’d wanted to, I’d have bought one, but having it on hand, at least you don’t have that barrier, you’ve got it on hand and that’s it. It’s like an extra support, in fact. (Patient 5 intervention group, achieved complete cessation, follow-up completed)

Some patients who did not have this direct access reported difficulties. Patient 6, from the standard care group, when asked if receiving treatment during the appointment would have helped her to stay on treatment, stated:

Oh well, completely, it would have been much easier. I would have had them. I would have had them at home and I certainly would have tried […], and I think I would have been less scared. It would have engaged me straight away (Patient 6, standard care group, treatment discontinued, no reduction or cessation).

### Carbon monoxide breath test monitors

Thanks to CO monitors, patients became aware of their consumption through the auditory and visual cues emitted by the device, highlighting smoking as a significant health problem requiring intervention. The testers allowed patients to visualize their cigarette consumption and level of intoxication in numerical terms, allowing for an objective assessment of their smoking behaviour:

‘Yes. In fact, I didn’t use it before the STOP program, and I was wrong, because in fact, I realized by using it, it had a really important impact to realize visually the intoxication we had, if I put myself in the patients’ shoes […]’(Doctor 2, GP)

This measurement tool can also provide clear evidence of patient progress and improvement. The numerical tracking of smoking cessation progress was highlighted by some patients as a motivational factor:

The doctor makes me blow into something to see where I’m at. Last time it was 15, while the one before that was 16. It’s still red, you see. But next time I’ll surprise her and it’ll be yellow. And until it turns green. (Patient 8, intervention group, reduced consumption follow-up ongoing)

### Interpersonal factors

#### Shared decision-making

SDM was identified as crucial by patients and HP. This approach allowed for the assessment of patients’ experiences, challenges, needs, and personal preferences:

Then, what can promote the doctor-patient relationship? In my opinion, it’s probably a model that’s not too paternalistic, a model where decisions are shared, where the issues are explained and shared, and so it’s the patient who takes ownership of the problem (Doctor 2, GP).

Some patients appreciated being able to actively participate in their treatment, which considered their experiences and individual circumstances. They reported that this involvement gave them a sense of autonomy and active participation in the smoking cessation process. Furthermore, the medical support and validation of their decisions positively influenced their treatment progress:

I was thinking about it, the commitment, what suited me, the choice she proposed, and then I got all the positive and negative information. And I felt more like I’d learned… When I made my choice to vape, I had the confidence, all the medical information she gave me, the trust, our choices, the extension of my choice… And that’s why it really took off for me, the trigger to stop. (Patient 4, intervention group, achieved complete cessation, follow-up completed)

### Recognizing of tobacco use as a disease

Perceiving smoking as a disease rather than a matter of willpower was identified as crucial for some patients to accept the idea of quitting within a medical framework. In this regard, HP explanations of addiction were essential, using scientific language adapted to patients’ understanding of addiction mechanisms and dependence.

And then, his [the HP] explanation of addiction, of the phenomenon of addictology, to understand at some point that it’s not just weakness, but that it can also be a disease to find yourself addicted to something […] (Patient 3, standard care group, achieved complete cessation, follow-up completed)

This process helped patients identify and anticipate the challenges they might face. HP explained the complexities of the cessation process, including potential withdrawal effects such as cravings and the risk of relapse. The goal is to anticipate these challenges and integrate them into the treatment framework and progression so that they are not seen as demotivating factors that could lead to setbacks or guilt, but rather as inherent symptoms of addiction.

[…] I present it as a chronic illness, and it’s precisely the illness of loss of control, what they call lack of willpower. I call it loss of control. So I’m also changing the way they talk. […] They need to feel less guilty, and that’s important. (Doctor 1, GP).

### Awareness of the need for a specific type of treatment

During medical appointments, HP provided detailed information about available treatments, explained their mechanisms of action, and addressed any negative perceptions or previous unfavourable experiences related to NRT or other methods.

When patients had negative experiences with previous treatments, HP clarified how these treatments worked and compared them to other medications. Some participants reported that this process helped them understand the importance of proper dosing to avoid adverse effects and maximize treatment effectiveness. Additionally, HP emphasized the importance of consistency and strict adherence to the treatment regimen, explaining that following the prescribed dosage and instructions significantly increases the chances of successful quitting while minimizing side effects.

And then, we take the whole history of what they’ve done, stopped, not stopped, what may have worked, what may not have worked. After that, we’re careful, because patients often tell us: ‘I’ve already tried to quit with my GP (or with anyone else) and patches don‘t work’. So why not? Because when you teach yourself, they only use one patch, and as I see it, you need several. So we’ll explain, we’ll actually say: ‘I told you to under-dose, that’s why it didn’t work.’ We can try again, at the right dose, and so on. […] (Doctor 3, tobaccologist)

The goal is to integrate available treatments into a medical framework, in which ongoing education and NRT are recognized as legitimate and controlled treatments. This will ensure their effectiveness, provided they are supported by continuous medical monitoring and patient adherence.

Ah yes, reassure them, show them that it’s not…. it’s not drugs, it’s just a treatment to help them at some point to quit smoking, and that they’ll be able to cut down and stop […] (Nurse 1)

This process focused particularly on the use of NRT, which was initially not considered a medical treatment by some participants. Integrating it into a medical framework is critical to maximizing its effectiveness.

I thought it was like smoking, actually [the electronic cigarette]. So I thought, well, cigarette or vaporizer, it’s the same thing, so I might as well keep cigarettes. But not at all […] But I thought the electronic cigarette was the same thing as cigarettes, but it’s not. (Patient 7, intervention group, reduced consumption; follow-up ongoing)

## Discussion

### Main findings

This qualitative study found that HP and patients alike valued several parts of the STOP smoking cessation programme. The following features have been identified as beneficial: extended consultation time, structured follow-up, free access to NRT and CO monitoring, and a collaborative approach to care.

#### Interpretation

Extended GP consultations were positively perceived by both HP and their patients, as they offered time for listening, explanation, and personalized treatment. This is especially relevant for smokers with low SEP, who often face barriers including fear of judgement, fear of failure, and limited health literacy (Okuyemi *et al.*, 2007). These findings align with other studies suggesting that tailored and accessible interventions are key to motivating smokers to engage in cessation services (Casetta *et al.*, 2017).

Moreover, extended medical appointments appeared to strengthen the GP-patient relationship. Patients can therefore feel more comfortable and less rushed, which encourages open dialogue (Jabour, 2020). However, sustaining longer consultations is challenging in the French healthcare system (as in other healthcare systems), where shortages of practitioners, particularly in underserved regions, and heavy workloads already strain capacity (Chevillard, Lucas-Gabrielli and Mousques, 2018). The requirement to consult a GP before accessing many specialists adds further complexity (l’Assurance Maladie, 2025).

Regular patient follow-up was also seen as highly beneficial. Patients valued continuous support, which provided consistency, allowed for timely adaptation of treatment, and reinforced motivation. This aligns with results from a randomized clinical trial showing that personalized follow-up, combined with face-to-face counselling, telephone support, and medication, increases the chances of quitting smoking (Fu *et al.*, 2014). Our results suggest that follow-up not only sustains engagement but also allows HP to identify and address other health issues and tailor care accordingly.

Direct and free access to cessation aids was also positively perceived. Financial and logistical barriers are well documented as obstacles for smokers attempting to quit, especially for smokers with low SEP (Roddy *et al.*, 2006). Evidence from the United Kingdom shows that providing free NRT in deprived areas improves quit rates and reduces consumption (Copeland, Robertson and Elton, 2005). Thus, barriers to accessing services and treatments can be a decisive factor in initiating and maintaining treatment. While economic barriers are frequently highlighted in the literature (Murray *et al.*, 2009; Stewart *et al.*, 2014), our study adds that immediate access to cessation aids and support could further enhance the chances of quitting. Removing waiting times reduces additional hurdles, such as pharmacy visits, which may expose patients’ smoking status and attempts to quit, and can reinforce stigma or self-doubt.

CO monitoring was particularly appreciated for offering visible, real-time feedback. Patients perceived it as a motivating tool that made progress tangible. Prior studies confirm that CO monitors can improve cessation outcomes when embedded in structured programmes (Shahab, West and McNeill, 2011; Vasthare, Kumar and Arron, 2018). They also provide an objective measure of smoking status, which can complement self-reports and guide clinical decisions (Baxter, 2016). Although their use on disadvantaged populations has not been extensively studied, our findings suggest that they may help motivation and adherence in these groups.

Another important finding concerns how smoking was framed during consultations. When described as a chronic condition rather than a matter of willpower, patients reported feeling less guilty and more motivated to persist despite relapses. This approach echoes broader shifts in the perception of smoking, from a socially accepted practice to a recognized medical condition requiring treatment (Rooke, 2013). Framing smoking in this way also helps clarify the role of nicotine replacement therapy and e-cigarettes, which some patients initially regarded with scepticism (Copeland, Robertson and Elton, 2005). Yet, some participants struggled to embrace this medical framing, which highlights the importance of clear and empathetic communication from HP. This is especially relevant for smokers with low health literacy, who are more prone to relapse after cessation treatment (Stewart *et al.*, 2014).

Finally, SDM seemed to encourage stronger patient engagement. Allowing individuals to select treatments that matched their preferences and values fostered a greater sense of control. At the same time, SDM appeared to build trust and improve the therapeutic relationship. This is consistent with evidence showing that SDM improves knowledge, risk perception, comfort with decisions, and adherence (Shay and Lafata, 2015). Importantly, SDM can also mitigate health disparities by using accessible language and formats that make information understandable for disadvantaged groups (King, Eckman and Moulton, 2011). Our findings suggest that SDM holds promise as a strategy to enhance both equity and effectiveness in smoking cessation care.

### Limitations

Our study has several limitations which need to be acknowledged. One potential limitation of this study is the relatively small number of interviews conducted. Although saturation was reached, the recruitment process may have introduced selection bias. To mitigate this, we employed a purposive sampling strategy to ensure participant diversity, especially in terms of smoking status. Moreover, we did not capture the full diversity of HP involved, and there was an over-representation of women (*N* = 14) compared to men (*N* = 5) among participants. Additionally, the fact that participants agreed to participate may introduce bias, as those unsatisfied with their treatment are more likely to decline participation. To limit these biases, we employed purposive sampling to ensure a diverse sample in terms of sociodemographic characteristics, smoking habits, and HP roles.

Another limitation pertains to the material challenges encountered when conducting the study interviews. Telephone interviews, in particular, lack important non-verbal cues and can be subject to technical difficulties, such as connection problems, potentially affecting the depth and quality of the data collected.

Another limitation relates to the use of observational notes. While these notes were valuable for informing and interpreting interview data and for enabling immersion in the field and validation of interpretations, they were not comprehensive enough to be used as an independent source of data in their own right. Their primary purpose was to complement the interviews rather than illustrate or analyse observations in their own right.

Finally, another potential limitation is the timing of the study, as some patients completed their treatment more than a year before their interview, which can impact memory and event reconstruction. Moreover, due to the sensitive nature of the topics discussed, certain elements may have been omitted due to distrust or embarrassment. However, we believe that smokers typically remember relevant information about why and how they quit. Nonetheless, it is important to acknowledge the potential presence of bias. Despite these limitations, this research provides a valuable theoretical guide for developing smoking cessation interventions based on the STOP trial.

## Conclusion

This study shows that both patients and HP viewed specific treatment features and interpersonal factors as central to the success of smoking cessation interventions for individuals with low SEP. Extended consultations, regular follow-up, free and immediate access to treatments, and the use of CO breath monitors were seen as supporting adherence, while shared decision-making, empathetic communication, and framing tobacco use as a disease reinforced trust and engagement. These findings have significant implications for smoking cessation programmes and policies. They highlight the need to improve treatment accessibility, strengthen provider training in patient-centred communication and allocate sufficient resources for regular follow-up, particularly in underserved populations.

## Data Availability

The data that support the findings of this study are available from the corresponding author upon reasonable request.
